# Petrological footprints of the millstones of Megara Hyblaea (Sicily Island, Italy) highlight the human interactions with Mediterranean volcanoes

**DOI:** 10.1038/s41598-022-16784-1

**Published:** 2022-07-21

**Authors:** P. Santi, C. Chaigneau, A. Renzulli

**Affiliations:** 1 Dipartimento di Scienze Pure e Applicate, Università degli Studi di Urbino Carlo Bo, 61029 Urbino, Italy; 2 Université Paris 1 Panthéon-Sorbonne, UMR 7041 ArScAn, Paris, France

**Keywords:** Environmental social sciences, Materials science

## Abstract

A petrographic and geochemical study of several volcanic millstones, representative of 119 artifacts found in the ancient Greek colony of Megara Hyblaea (Sicily Island) and recording the grinding device evolution from the Archaic to the Hellenistic period, unravelled the volcanoes involved as quarrying and production areas. This was possible also through the comparison with available petrographic and geochemical literature data of ancient volcanic millstones found in the whole Mediterranean. Saddle querns, hopper-rubber, rotary Morgantina- and Delian-type millstones of Megara Hyblaea consist of lithotypes belonging to five magmatic series: Tholeiitic, Na-Alkaline, Tholeiitic Transitional, Calcalkaline and High-K Alkaline. A provenance from the Eastern Sicily, i.e. mugearites from Etna and basalts and basaltic andesites from the Hyblaean Mountains were recognized for all the four investigated grinding devices. By contrast, a sea-trade is involved for several saddle querns made of calcalkaline basaltic andesites and andesites lavas (Aegean Islands) and two Morgantina-type millstones consisting of a calcalkaline rhyodacite ignimbrite from the quarrying site of Mulargia (Sardinia). A wide millstone trade, both local (Eastern Sicily) or maritime (Central-Eastern Mediterranean) was thus constrained through six centuries, from the foundation of the Greek colony up to the destruction of the settlement at the end of third century BCE. Finally, Vulture Volcano (southern Italian peninsula) is the most probable candidate for the only leucite- and haüyne-bearing phonolite of the High-K Alkaline Series.

## Introduction

**Figure 1:**
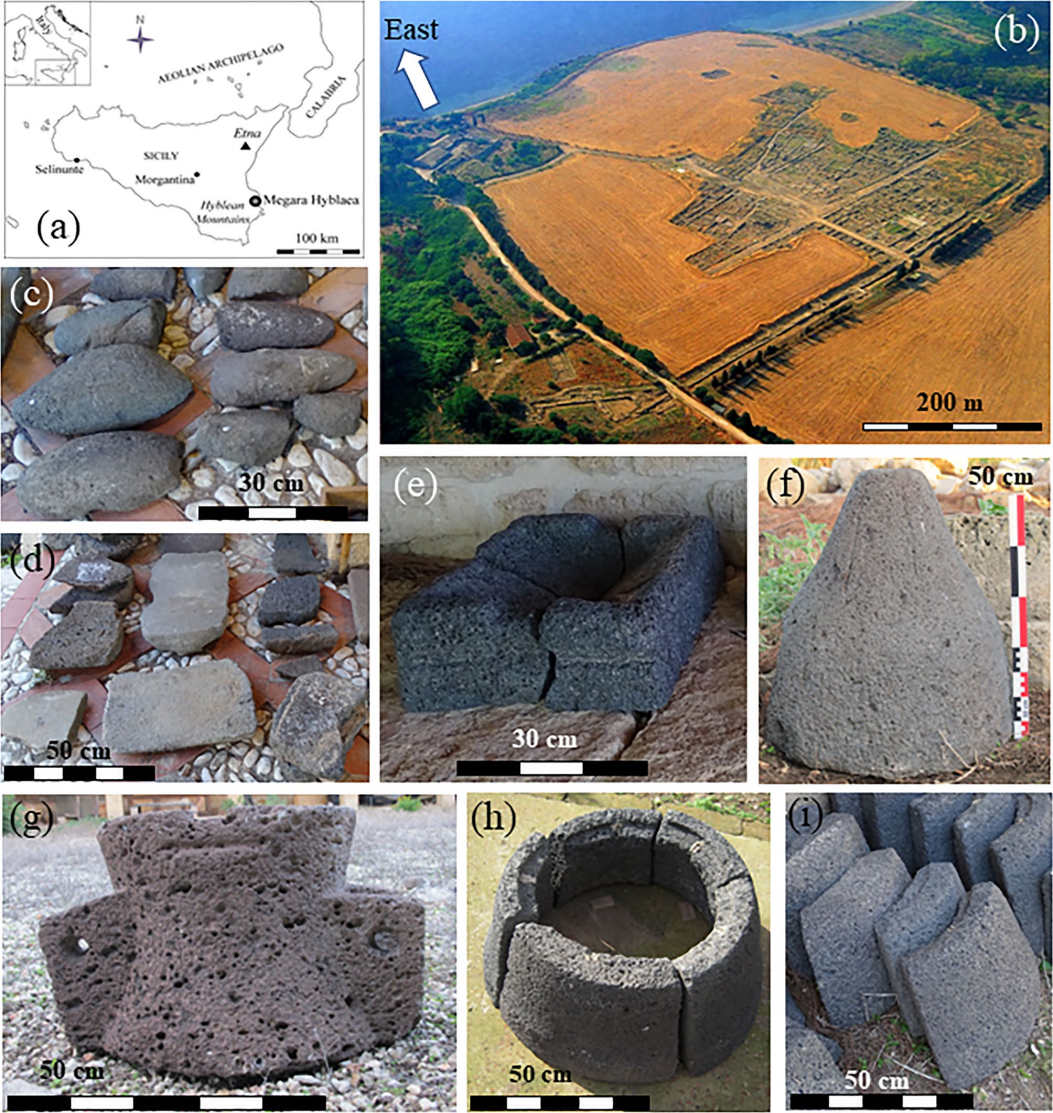
Location of Megara Hyblaea in the Sicily map (**a**) and aerial view of the archaeological site (**b**). Representative millstones: saddle-querns upper (**c**) and lower (**d**) portions; rectangular hopper-rubber (**e**); rotary Morgantina-type *meta* (**f**) and *catillus* (**g**); assembled *catillus* (upside down) of a composite Delian millstone (**h**) and relative isolated elements (**i**).

Megara Hyblaea is one of the oldest Greek colonies in Sicily. It was founded in 728 BCE on the eastern coast of the island (Fig. 1a,b). Since 1949 the exploration of the site is conducted as part of an agreement between the Soprintendenza della Sicilia Orientale and the École Française de Rome. Megara Hyblaea underwent two major phases of settlement^1,2^ one in the Archaic period (eighth-fifth century BCE) and the other in the Hellenistic one (fourth-third century BCE). Although the above two flourishing periods both ended with a brutal destruction, the excavations have shown that the site was inhabited almost continuously until the fourth century AD^3^. The huge collection of grinding stones found at Megara Hyblaea consists of 123 millstones for cereals of which 119 made of volcanic lithotypes (Fig. 1c–i) including saddle-querns, hopper-rubber and Morgantina-type millstones, and elements of composite millstones also known as Delian-type. Four manual rotary millstones made of sedimentary rocks and corresponding to the late Roman sporadic settlement of the site^4^ are beyond the aim of the present work. Here we deal with the petrographic and geochemical study of 57 representative samples among the volcanic artifacts. According to the grinding technology evolution^5–10^ these millstones therefore cover the Archaic, Classic and Hellenistic periods of Megara Hyblaea which is definitively the most important millstone case study for Greek Sicily.

Saddle-querns, the oldest type of grinding device in the Mediterranean, used since the Late Paleolithic^11^ are the most represented at Megara Hyblaea (80 millstones, undamaged, partially broken or fragments; Fig. 1c,d; Fig. S1a), consisting of upper stones of various shapes (oval, ovoid, quadrangular, or irregular) and thick oval or quadrangular lower stones. A standardized subtype (Fig. S1a) combines elongated boat-shape upper stones with a concave grinding surface and rectangular lower stone tables^4,10,12^.

In the early fifth century BCE, a major innovation on the grinding device led to the manufacturing of the hopper-rubber millstones, also called *Olynthian* mills^9^ which were used until the Roman period^10^. First, the upper part was equipped with a central cut, the hopper, allowing the grain to be distributed down to the basal portion during the grinding process. This new grinding device operated on reciprocal motion, manually, as did the saddle querns^5,8^. This is shown by one hopper millstone at Megara Hyblaea^4^, oval and equipped with a slot (MH53; Fig. S1b). Second, the addition of a simple mechanism based on the principle of the lever allowed an amplification of the movement of the upper stone and a standardized type of the hopper-rubber millstone was then developed with a rectangular upper stone equipped with cuttings for fixing a wooden handle (Fig. 1e; Fig. S1b). In Sicily and the eastern Mediterranean, these mills worked in an arched back-and-forth motion^13,14^. The ten well-preserved or damaged hopper-rubber millstones found at Megara Hyblaea belong to a Sicilian sub-type because of their morphology (shape of the hopper and specific cuts for fixing the handle; Fig. S1b)^4^.

Meanwhile medium-sized rotary mills appeared in the central Mediterranean: the Morgantina-type millstone. The lower motionless lower stone (*meta*, Fig. 1f; Fig S1c) is simply conical and the upper stone (*catillus*; Fig. 1g; Fig. S1c) is slightly-biconical with the upper truncated cone (serving as a hopper) also having two “ears-like” elements where the wooden handles were fixed, allowing one or two men to operate the mill by walking around it^6^. The name of these millstones derives from the archaeological site of Morgantina, near Enna (Sicily) where they were found in large numbers^6,15^. This grinding device is found only in the central Mediterranean (Mallorca, Sardinia, Sicily, North Africa) that is, mainly in the areas closest to the Punic sphere^6,10,16^. It appeared in the early fourth century BCE and it was still in use in Sicily until at least the second century BCE before it was replaced by the larger, perfectly biconical (hourglass-like) millstones, the so-called Pompeian mill^6,8,10,14^. The widespread use of the Morgantina-type rotary millstones in the Greek Sicily well agrees with a period of prosperity in the eastern sectors of the island, due to the relatively peaceful reign of Hieron II of Syracuse (275–215 BC)^17^ providing an ideal context for the invention of this more efficient rotary mill. Eleven well preserved to partially broken Morgantina-type millstones were found in Megara Hyblaea (comprising both *metae* and *catilli*). One *meta* (MH113; Fig. 1f) was found in a Hellenistic house established to be as early as the third century BCE and a base suitable for such a mill is also present in a bakery from the first century BCE^3,4^.

A great peculiarity of the Megara Hyblaea grinding tools collection is the presence of 18 elements of composite millstones (Fig. 1h,i; Fig. S1d) belonging to eight mills. This grinding device is sometimes referred to as Delian-type mill, after the Delos Island (Aegean Islands, Greece), where it is largely predominant^18–20^. This rotary mill consists of two annular millstones constituted by 5 to 7 blocks fixed by means of a large wooden and metal frame. The segmentation would allow for larger mills to be made when it was not possible to extract blocks of these sizes. They were identified only on Greek sites throughout the Mediterranean. They have been using at least from the beginning of the second century BCE (at Ephyra, Greece) to the fourth century AD (at Badia, Egypt)^21^.

There are plenty of volcanoes in the Mediterranean and the petrographic and chemical investigations of the ancient volcanic grinding tools are challenging to trace the trade networks of the millstones that accompanied the human interactions. Igneous petrology applied to the study of volcanic millstones in terrestrial archaeological sites or shipwrecks is a powerful tool to define the volcano or the volcanic areas where lavas (or pyroclastic rocks) were exploited to make grinding tools^14,16,22–25^. In this framework the huge millstones collection of the Greek colony of Megara Hyblaea represented, for the long-time of the settlement and the occurrence of four grinding devices (saddle-querns, hopper-rubber millstones, and rotary Morgantina and Delian-type composite millstones), a chance to follow the evolution of the grinding techniques and recognize the provenance of volcanic rocks used for manufacturing millstones and relative human interactions.

## Sampling and methods

The sampling in the archaeological site of Megara Hyblaea was performed, for the majority, on fragments (maximum two cubic centimetres each) of already partially broken millstones, because only in few cases there are complete artifacts. On the basis of the different shape reflecting the evolution of the grinding technology, we selected 57 samples of volcanic millstones to be studied through thin section mineralogy and petrography, representative of four grinding devices: (1) thirty-one fragments coming from saddle-querns (eight basal flat portions and twenty-three upper mobile elements); (2) six fragments of hopper-rubber millstones; (3) eleven samples from rotary Morgantina-type millstones (six *metae* and five *catilli*) and (4) nine fragments of single elements of the rotary composite Delian-type millstones (Table S1; Fig. 1). Thin section petrography using polarizing optical microscopy was carried out to characterize the modal mineralogy and textures of all the 57 samples. Whole-rock chemistry was determined at the Activation Laboratories LTD (Ancaster, Canada) by ICP-OES (Inductively Coupled Plasma-Optical Emission Spectrometry; Varian Vista 735) and ICP-MS (Inductively Coupled Plasma-Mass Spectrometry; Perkin Elmer Elan 9000) for major (wt%) and trace elements (ppm) respectively. Samples were crushed and powdered in an agate mortar to avoid contamination as much as possible and fused by lithium metaborate/tetraborate technique in an induction furnace, providing a fast and high-quality fusion. The resulting molten bead was rapidly digested in a weak (5%) nitric acid solution containing an internal standard and mixed continuously until completely dissolved. It is only with this attack that major oxides including SiO_2_, refractory minerals (i.e. zircon, sphene, chromite, etc.), REE and other high field strength elements are put into solution. Calibration was performed using 14 prepared USGS and CANMET certified reference materials. One of the 14 standards is used during the analysis for every group of ten samples. Errors, calculated using the certified natural rock standards and replicates of some samples, are generally < 2% and < 5% for major oxides and trace elements respectively. Detection limit for each analysed element is shown in Table S2.

Comparing thin section study (57 samples) and major-trace elements chemical analyses (46 samples out of 57) we defined both volcanic rock classification and magmatic series of all the samples (Table S1).

The majority of the studied artifacts are made of grey, dark-grey to black lavas with different degree of vesicularity and porphyricity. Only two fragments of *catilli* of Morgantina-type millstones consist of a reddish highly vesiculated pyroclastic rock (ignimbrite).

## Results

### Chemical classification and petrographic characterization

**Figure 2:**
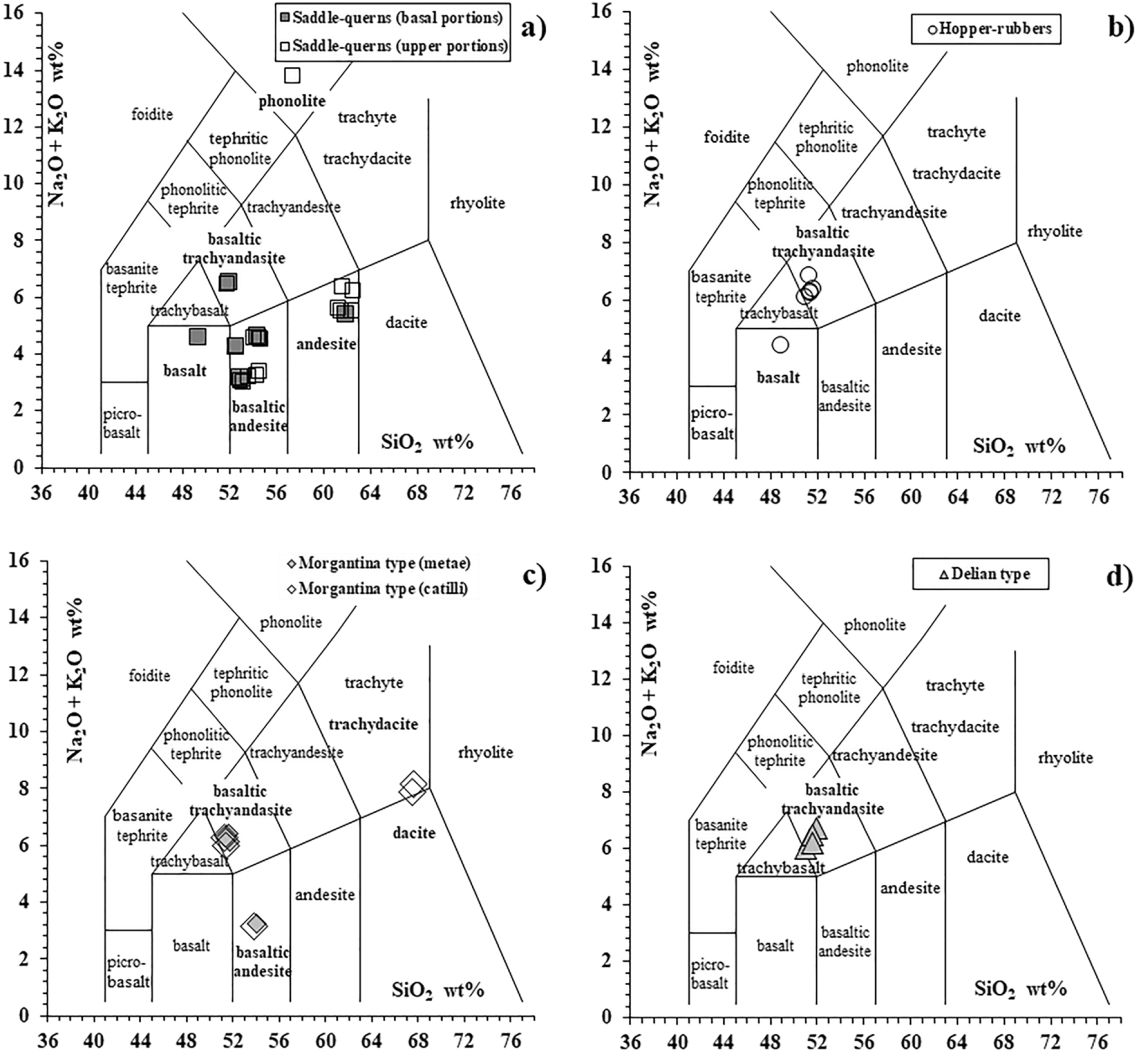
The Total Alkali-Silica classification diagram for volcanic rocks^27^; (**a**) saddle-querns; (**b**) rectangular hopper-rubbers; (**c**) rotary Morgantina-type; (**d**) Delian-type.

According to the IUGS recommendations^26^, for a correct petrographic classification of the studied volcanic millstones we used the total alkali (Na_2_O + K_2_O) versus silica (SiO_2_) diagram (TAS^27^; Fig. 2). According to the Na_2_O and K_2_O contents we consider the basaltic trachyandesites as mugearites (Na-Alkaline) as they have Na_2_O minus 2.0 ≥ K_2_O^26^. The overall major and trace elements chemical data of 46 millstones are reported in Table S2.

**Figure 3:**
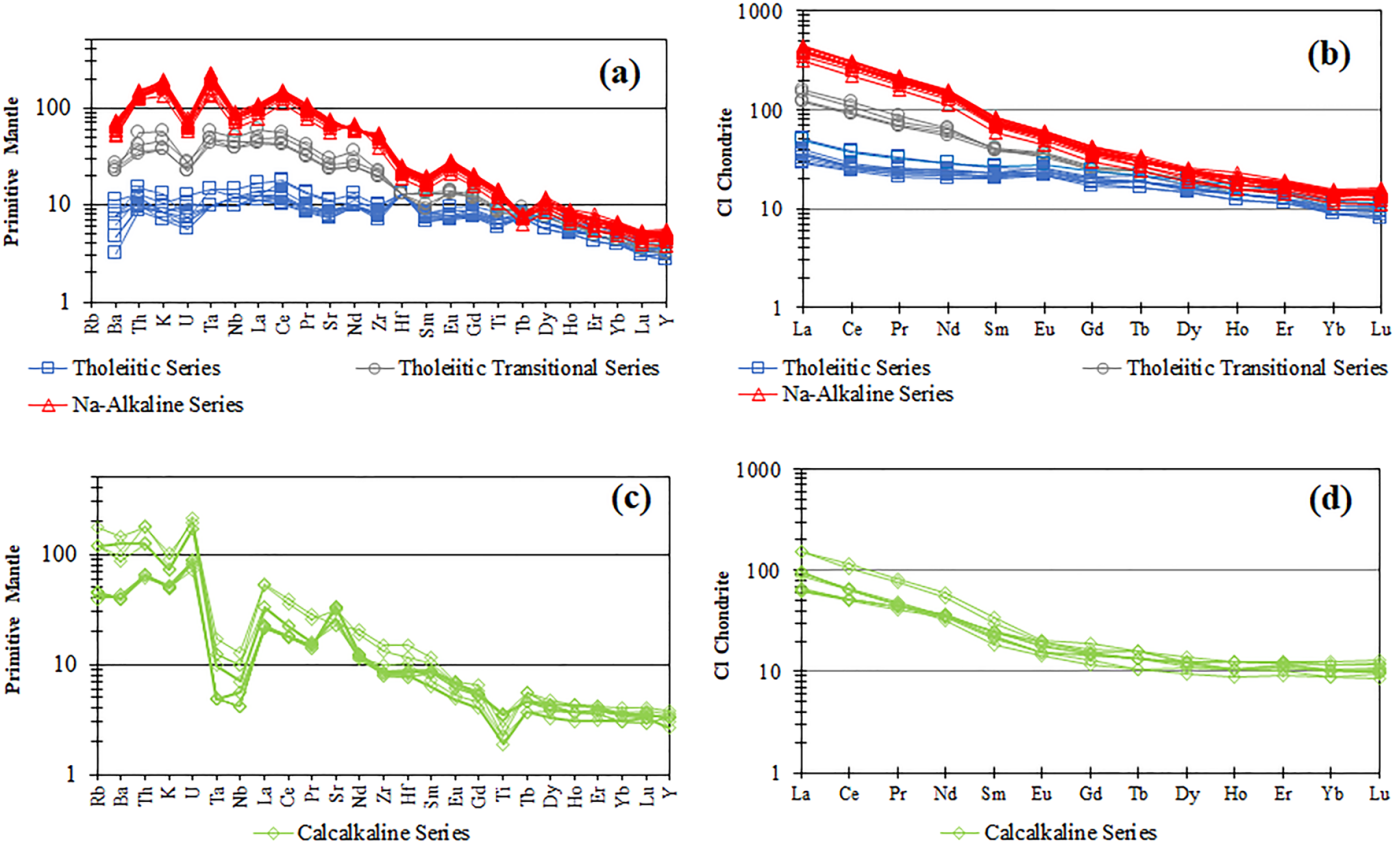
Incompatible trace elements patterns normalized to Primitive Mantle (**a**,**c**) and Rare Earth Elements patterns normalized to CI Chondrites (**b**,**d**) according to Sun & McDonough^28^. The millstones belonging to the different magmatic series are reported in Tables S1 and S2. The two rhyodacite ignimbrite samples (Calcalkaline Series) and the phonolite (High-K Alkaline Series) are not reported in these diagrams.

The saddle-querns show a widespread compositional range (Fig. 2a) comprising one basalt, basaltic andesites, andesites, mugearites and one phonolite, whereas the other typologies of grinding stones are mainly mugearites, with the exception of one hopper-rubber basalt, and, for the Morgantina-type millstones, two basaltic andesites and two rhyodacite ignimbrites (Fig. 2b,c). Only the Delian-type mills are completely homogeneous in composition being all represented by mugearites (Fig. 2d). Incompatible trace elements and Rare Earth Elements of all the chemically investigated millstones, normalized to Primitive Mantle and CI Chondrites respectively (Fig. 3), show the presence of distinct patterns of magmatic series. According to the fundamental trace elements abundances of volcanic rocks associated to the geodynamic environments^29,30^ Tholeiitic to Tholeiitic Transitional MORB-like (Middle Ocean Ridge Basalts) and Na-Alkaline OIB (Ocean Island Basalts) patterns are defined by Fig. 3a, showing sub-parallel trends characterized by relative depletion of HFSEs and LILEs for the first two series and a high enrichment for the incompatible elements for the Na-Alkaline Series. Also, the REE patterns of these samples are sub-parallel (Fig. 3b) with a well-defined enrichment of the Light and Middle Rare Earth Elements passing from the Tholeiitic to the Na-Alkaline Series, emphasized by the increasing slope of the patterns (i.e. higher LRREs/MREEs ratios). The millstone samples belonging to the Calcalkaline Series are pointed out by the incompatible elements patterns with negative Ta, Nb and Ti anomalies (Fig. 3c) which are very distinctive features of subduction-related rocks (volcanic arcs). Different enrichments in incompatible trace elements and REEs (Fig. 3d) of the calcalkaline millstones are simply due to the different degree of magma evolution of the different lithotypes (basaltic andesites vs. andesites). Using principles of parameter divides of the TAS diagram field, it is also inferred that also the two rhyodacite ignimbrite millstones (Fig. 2c) belong to the Calcalkaline Series whereas the phonolite lava clearly matches with a silica-undersaturated High-K Alkaline Series (Fig. 2a). The five magmatic series, detected by geochemical data, are also confirmed by the thin section modal mineralogy and petrography which will be described below.

#### Mugearites (Na-Alkaline Series)

They are represented by 23 porphyritic lava millstones (Table S2; Figs. 2, 3a,b) with a Porphyritic Index (PI) between 15 and 30 vol% and an intergranular to pilotassitic microcrystalline groundmass (Fig. S2a,b). Mostly rounded vescicles may reach 10–15 vol%. Modal mineralogy, both as phenocrysts and groundmass, is mainly constituted by plagioclase and clinopyroxene. Olivine is also present as a fundamental mineral whereas opaques represent the main accessory phases. Secondary calcite crystals may occur.

#### Basalts (Tholeiitic Transitional Series)

The two basalts (Table S2; Figs. 2a,b, 3a,b) show different textures. MH104 is a doleritic sub-aphyric lava with a PI ≤ 1 vol% and a vesiculation of 5–7 vol% with some secondary phases (mainly calcite) filling the vesicles. The rare phenocrysts and the groundmass are represented by oxidized olivine, plagioclase, clinopyroxene and opaques minerals consisting of rounded grains of magnetite and laths of ilmenite. Fine-grained clinopyroxenite cumulates/xenocrysts are also detected. By contrast MH53 basalt (Fig. S2c) is a porphyritic lava (PI 5–7 vol%) with a microcrystalline intergranular groundmass and a vesiculation of about 3–5 vol%. The fundamental mineral assemblage is constituted by olivine, plagioclase and clinopyroxene whereas opaque rounded grains are the accessory phases.

#### Basaltic andesites (Tholeiitic, Tholeiitic Transitional and Calcalkaline Series)

They can be distinguished by chemical composition (Table S2; Fig. 3). As concerning the thin section petrography, the tholeiitic basaltic andesites (14 samples) are represented by doleritic microcrystalline nearly aphyric lavas, with an intergranular groundmass. A relatively high vesicularity (10–25 vol%), at places containing secondary calcite crystals is very common. The modal mineralogy consists of plagioclase laths, pale green clinopyroxene, more or less oxidized olivine and sub-rounded grains and laths of opaque minerals (magnetite and ilmenite) as accessory phases (Fig. S2d,e). The two Tholeiitic Transitional basaltic andesites (Fig. S2f) are constituted by a microcrystalline intergranular groundmass with a PI of about 5 vol% and vesiculation of 3–5 vol%. The mineralogical assemblage is represented by plagioclase, clinopyroxene, skeletal oxidized olivine and opaques minerals as rounded grains or laths. As described for the doleritic basalt (MH104), also in these samples fine-grained clinopyroxenite cumulates/xenocrysts are present. The five basaltic andesites of the Calcalkaline Series (Fig. S2g) are represented by porphyric-seriate lavas (PI 15–20 vol%) with a microcrystalline groundmass, at places intergranular to pilotassitic and vesicles around 5 vol%. The phenocrysts consist of well-developed plagioclases with, at places, concentric zonation, and pale green clinopyroxene with inclusions of opaque minerals. The same mineral assemblage constitutes the groundmass where opaques are also present. Their K_2_O/Na_2_O ratios are between 0.48 and 0.51 (Table S2).

#### Andesites (Calcalkaline Series)

These eight samples (Table S2; Fig. 3c,d) are represented by highly porphyritic lavas (PI 25–35 vol%) with micro-cryptocrystalline to glassy groundmass (Fig. S2h). The vesiculation is relatively low (about 2–5 vol%). Phenocrysts consist of large horneblende and zoned plagioclase at places with sieve texture (and partially replaced by sericite and clay minerals), pale green clinopyroxene and rounded opaque minerals*.* K_2_O/Na_2_O ratios range from 0.64 to 0.95 (Table S2).

#### Rhyodacites (Calcalkaline Series)

They consist of two millstones (Table S2; Fig. 2c) characterized by a vitrophyric texture with a glassy fluidal groundmass (Fig. S2i) thus emphasizing a pyroclastic rock (ignimbrite) rather than a lava. The abundant vesicles (25–30 vol%) show an internal rim bordered by green celadonite and other secondary minerals. The partially broken phenocrysts/xenocrysts mainly consist of K-feldspar (sanidine), zoned plagioclase and pyroxene.

#### Phonolite (High-K Alkaline Series)

This sample (Table S2; Fig. 2a) is represented by a porphyritic lava (IP 10 vol%) with a microcrystalline groundmass. Micro-vesiculation is low. The modal mineralogy consists of feldspathoids (leucite > > haüyne), K-feldspar (sanidine), plagioclase, green zoned and pleochroic clinopyroxene with biotite inclusions. Magnetite and titanite are the main accessory phases (Fig. S2l).

## Discussion

The millstone trade in antiquity throughout the Mediterranean, at least from the Bronze Age to the Romans, was emphasized since the pioneer works of the last four decades of the past century^22,23,31–34^ mostly dealing with artifacts in terrestrial archaeological sites where volcanic rocks were the most widespread as grinding stone tools because of their best performance to grind cereals with respect to other lithologies^8,10,11,35^. In addition, the key-role played by studying millstones of shipwrecked cargoes was also pointed out, to define the trade networks from the volcano/volcanic area of provenance to final destinations^14,16,36,37^. Concerning the variability of volcanic rocks throughout the Mediterranean region, linked to geodynamics and relative mantle melting processes, magmatic series can be roughly distinguished in two general groups, namely (1) anorogenic^38^ and (2) subduction-related^39–41^. In this way, petrographic and chemical comparisons of hundreds of volcanic millstones with potential volcanoes or volcanic areas throughout the Mediterranean led to recognize the main local or far-away sources of the volcanic grinding stones found in archaeological settlements^14,23,24,42^. These are mainly: (1) Italian volcanoes of the Roman Volcanic Province such as the Vulsini Volcanic Complex (with a quarrying site near Orvieto)^31,43–45^ and Vesuvius^46^ or Vulture Volcano^47,48^; (2) Eastern Sicily volcanoes (Etna and Hyblaean Mountains)^12,15,44,49,50^; (3) Sardinia volcanic provinces (mainly Mulargia site)^51^; (4) volcanic islands of the Sicilian Channel (mainly Pantelleria)^37,52^ or the Southern Tyrrhenian Sea (Aeolian Archipelago and Ustica Island)^49,53,54^; (5) volcanic islands of the Aegean Sea (mainly Nisyros)^16,23,55^. By contrast, lavas from the Levant Area (Lebanon, Israel, Jordan and Syria)^34^, Morocco (Middle Atlas)^23,56^, Libya (e.g. Gharyan area)^23^, Northeast Spain and France (e.g. Olot and Gerona; Massif Central, Agde)^23^, were mainly used as millstones for local use and not transported by long sea-trade throughout the Mediterranean.

**Figure 4:**
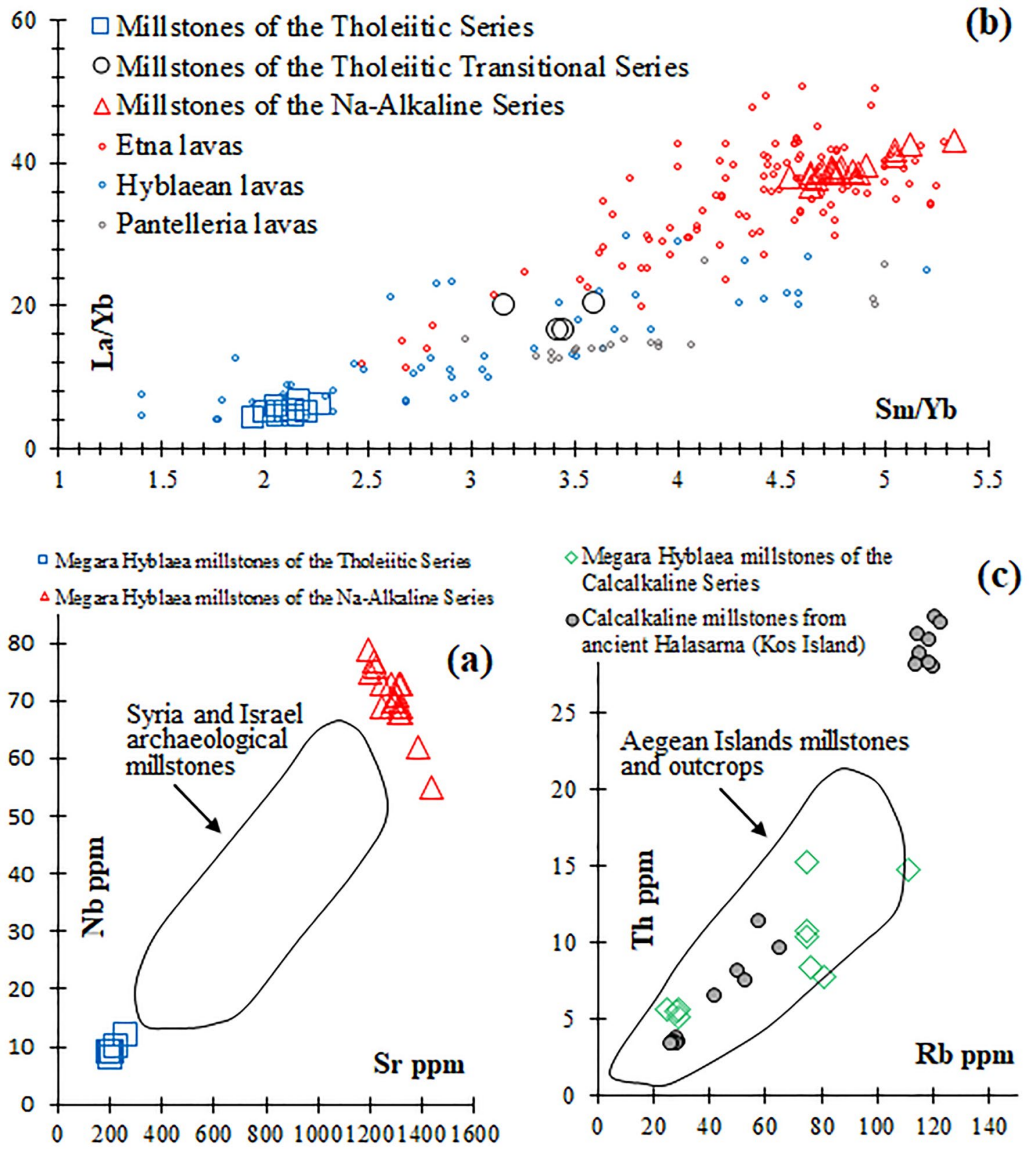
Nb vs. Sr (**a**), La/Yb vs. Sm/Yb (**b**) and Th vs. Rb (**c**) diagrams comparing Megara Hyblaea millstone compositions with literature data. (**a**) Field of archaeological millstone data from Syria and Israel^34^; (**b**) literature data of lavas with compatible mineralogy and major oxide composition from Etna, Hyblaean Mountains and Pantelleria^57^; (**c**) data of millstones coming from the ancient quarries of Kos and Nisyros Islands^55^ and field of Aegean Islands millstones and outcrops^33^.

The very high Sr contents (1191–1433 ppm; Table S2) of the mugearite millstones of Megara Hyblaea, coupled with Ni and Cr below the detection limit (< 20 ppm) define their provenance from the Na-Alkaline lavas of Etna Volcano, as confirmed by the literature data of the Mediterranean igneous grinding stones made of basic rocks (SiO_2_ 45–52 wt%)^16,34^. The Nb versus Sr diagram (Fig. 4a) clearly rules out, for the mugearite grinding stones of Megara Hyblaea, a provenance from the alkali olivine basalts of the Levant area used for millstones, whereas the La/Yb versus Sm/Yb diagram (Fig. 4b), definitively indicates, among the most exploited OIBs source areas for millstones of the Central Mediterranean (Etna, Hyblaean Mountains and Pantelleria Island), a provenance from the Etna Volcano. Figure 4b also highlights a provenance from the Hyblaean Mountains of all the basaltic andesite millstones of the Tholeiitic Series. Low Sr and Nb, 202–268 ppm and 8–12 ppm respectively (Table S2) are also clear geochemical parameters to rule out for these millstones a provenance from the Levant basaltic source areas (Fig. 4a). In addition, relatively low Zr contents (84–98 ppm; Table S2) of the grinding stones of the Tholeiitic Series is against a provenance from Egypt as well (cf. Figures 16, TiO_2_ vs. Zr of Williams-Thorpe & Thorpe^34^). The two basalts and the two basaltic andesite millstones of the Tholeiitic Transitional Series, which are not well constrained in the La/Yb versus Sm/Yb diagram (Fig. 4b), have however relatively low TiO_2_ (1.65–2.05 wt%) and therefore cannot match Pantelleria basalts (TiO_2_ ≥ 2.4 wt%)^14,37,57^. For these four investigated millstones, the Hyblaean Mountains source area is also strongly suggested as they clearly match with the Tholeiitic Transitional Series of this Eastern Sicily volcanic province (cf. with the Tholeiitic Transitional lavas of the Hyblaean Mountains)^57^. This is also supported by the presence, in these stone artifacts, of some clinopyroxenite cumulates/xenocrysts which are common in the volcanic rocks of the Hyblaean Mountains^57^.

For the basaltic andesites and andesites millstones of the Calcalkaline Series (Table S2), clearly belonging to a subduction-related volcanic-arc environment, the two major volcanic source areas of Central-Eastern Mediterranean, known as quarrying sites for millstones in antiquity, are the Aegean Islands and the Aeolian Archipelago^16,32,49,55^. Among the Aeolian Islands, Lipari was a flourishing Greek colony (*Meligunis*)^58–60^ and hopper-rubber and rotary Morgantina-type millstones made of local High-K calcalkaline basaltic andesites and andesites were already discovered^49^. On the other hand, among the Aegean Islands known as millstone production centres, Nisyros represents the most famous quarrying site in the past, at least from the fourth century BCE^16,32,33,55^. The local calcalkaline millstones from Lipari have however a K_2_O/Na_2_O distinctively higher (0.75–1.00 for the basaltic andesites and 1.10–1.58 for the andesites)^49^ than the calcalkaline lava millstones of Megara Hyblaea (Table S2) and thus a provenance from the Aeolian Archipelago should be ruled out. K_2_O/Na_2_O ratios of millstone artifacts from Nisyros and Kos Islands, such as those found in the archaeological site of Ancient Halasarna (Kos Island, very close to Nisyros)^55^ range from 0.33 to 0.57 (basaltic andesites) and 0.59 to 1.04 (andesites), thus compatible with the calcalkaline grinding stones of Megara Hyblaea. In the Th versus Rb diagram (Fig. 4c) proposed by William-Thorpe & Thorpe^33^ for millstones of the Aegean Islands (both outcrops and archaeological samples) updated with data of millstones of the ancient Halasarna, the calcalkaline millestones of Megara Hyblaea are within the main compositional field of the Aegean volcanic islands inferred by the literature to have been exploited for millstones in antiquity.

The two Morgantina-type millstones consisting of a rhyodacite pyroclastic rock, are petrographically and geochemically compatible with the ignimbrite quarrying site of Mulargia (Sardinia) very widespread for manufacturing millstones which were found in several Mediterranean archaeological sites^22–24,51,61^. Reddish colour, eutaxitic/fluidal glassy texture, fundamental modal mineralogy, secondary phases (e.g. the presence of celadonite microcrystals bordering vesicles) and chemical composition clearly match with the well-known Sardinian millstone quarrying site of Mulargia^51^.

Finally, the phonolite saddle-quern (High-K Alkaline Series) showing a K_2_O/Na_2_O ratio of 2.1 (Table S2) rules out a provenance from the famous leucite phonolite quarrying site of the Roman Volcanic Province located near Orvieto (Central Italy, between Sugano and Buonviaggio, Vulsini Volcanic District) having K_2_O/Na_2_O ratio > 3.5^43,45^, one of the most exploited quarrying site for millstones from the Etruscan to the Roman Period and found in several archaeological sites throughout the Mediterranean^14,24,44^. Also, trace elements abundances of the Megara Hyblaea phonolite saddle-quern, such as Th (112 ppm) and Ba (945 ppm), rules out a provenance from this widely exploited leucite phonolite lava quarrying site from Central Italy (Th 150–178 ppm; Ba 2165–2313 ppm)^43^. Moreover, the presence of haüyne microphenocrysts in the phonolite saddle-quern of Megara Hyblaea definitively exclude the quarrying site near Orvieto, while strongly supports a provenance from the haüyne-bearing lavas of Vulture Volcano in Southern Italy which were also widely exploited as grinding stones starting from the Bronze Age^47,48^.

## Conclusions

**Figure 5:**
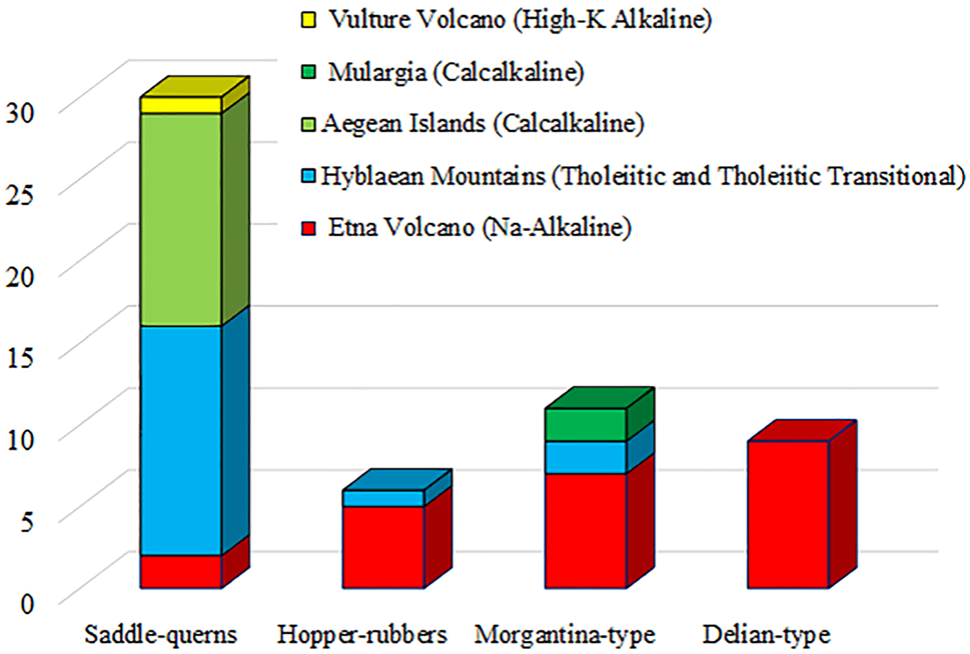
Quantitative distribution of the different millstone types of Megara Hyblaea with respect to their provenance areas and related magmatic series of the exploited volcanic rocks.

Source areas and relative petrological footprints (i.e. magmatic series) of the volcanic millstones found at Megara Hyblaea and distinguished on the basis of the different grinding devices, are reported in Fig. 5. Inhabitants of Megara Hyblaea quickly began to exploit the local lavas, using volcanic rocks from Etna and Hyblaean Mountains (Na-Alkaline mugearites, Tholeiitic and Tholeiitic Transitional basalts and basaltic andesites) to manufacture saddle-querns. The earliest chronological reference is provided by the saddle-quern MH37 discovered in a time context dated between 650 and 600 BCE, i.e. in the century following the foundation of the city (“Silos IIs 1949”^62,63^). Then, exploitation of lavas from quarrying sites in Eastern Sicily, through six centuries, from saddle-querns to hopper-rubber mills up to the rotary Morgantina and composite Delian-type millstones therefore emphasizes a robust terrestrial trade and local human interactions. Nevertheless, Megara Hyblaea was also connected with the flourishing Archaic to Hellenistic Mediterranean sea-trade, as shown by saddle-querns from the Aegean volcanic islands and two Morgantina-type millstones from the quarrying site of Mulargia in Sardinia. Millstones from the Aegean Islands would have been brought by the first settlers in the middle of the eighth century BCE or later on, as testified by the presence of the imported standardized boat-shaped saddle-querns. The maintenance of a local (Eastern Sicily) and extra-regional (Aegean) millstones supplying, more than a century after the foundation of the city, is confirmed by some saddle-querns originating from both areas (e.g. MH128-130) discarded with ceramics dated between 580 and 500 BCE (“well 2204”)^2^. The presence of only one leucite- and haüyne-bearing phonolite saddle-quern may have represented an occasional grinding stone (from Vulture Volcano) arrived to Megara Hyblaea accompanying goods or as a block collected as ballast in ships coming from the Adriatic coasts of southern Italy (Puglia-Basilicata). In the Archaic period, Megara Hyblaea was thus integrated into the properly Greek, eastward-facing trading routes whereas it seems not to have been involved in the Mediterranean millstone trade from the Punic area of influence (Pantelleria, Sardinia or southern Spain). In the late Classic and Hellenistic periods, a time of prosperity and economic flourishing for Sicily^17^, Etna Volcano became the main millstone production area for the Megara Hyblaea settlement for both the rectangular hopper-rubber millstones and the composite Delian-type mills which were massively manufactured with mugearites. This is also supported by the Morgantina-type millstones being mostly represented by the mugearites from Etna and subordinately by Tholeiitic basaltic andesites of the Hyblaean Mountains and the Mulargia rhyodacite ignimbrite from Sardinia. This latter provenance thus testifies a partial integration of Megara Hyblaea into the regional trade networks of the Central Mediterranean as well.

## Supplementary Information

Supplementary Information 1.

Supplementary Information 2.

## Data Availability

All data generated or analysed during this study are included in this published article (and its supplementary information files).
