# Supplementary material for: Petrological footprints of the millstones of Megara Hyblaea (Sicily Island, Italy) highlight the human interactions with Mediterranean volcanoes
Source: Sci Rep. 2022 Jul 21;12:12494. doi: 10.1038/s41598-022-16784-1 (PMC9304362; doi:10.1038/s41598-022-16784-1)
Supplement: Supplementary file 1 — Supplementary Information 1. [file 41598_2022_16784_MOESM1_ESM.pdf]

Figure S1 -Type and size of the millstones found at Megara Hyblaea: (a) saddle-querns (lower and upper parts); (b) hopper-rubber millstones (manual oval type and rectangular lever-worked type); (c) Morgantina-type mill (*meta* and *catillus*); (d) composite Delian-type mill (*meta* and *catillus*). Saddle querns comprise upper stones of various shapes (oval, ovoid, quadrangular, or irregular, between 21-49 cm long) and thick oval or quadrangular lower stones (between 45-57 cm long and 23-38 cm wide). Standardized subtype prevails consisting in an elongated boat-shape upper stones with a concave grinding surface (between 30-40 cm long) associated with rectangular grinding lower stones (between 45-57 cm long and 27-38 cm wide). Size of the rectangular hopper-rubber millstones is 40-50 cm long and 35-40 cm wide. *Metae* of the Morgantina-type millstones show a height between 40-65 cm and a maximum diameter (base) between 50-55 cm whereas *catilli* have a height between 40-45 cm and a maximum diameter of the grinding surface (lower cone) between 40-50 cm. The Delian-type composite millstones are large size rotary mills of 60-90 cm in diameter belonging here to a subtype specific to the central Mediterranean (Sicily, Adriatic Sea coasts) characterized by a height between 25-40 cm (compared to 17-35 in Delos), and 4 to 6 elements to form one millstone (compared to 6-8 in Delos) and a high degree of finishing of the non-grinding surfaces[4].

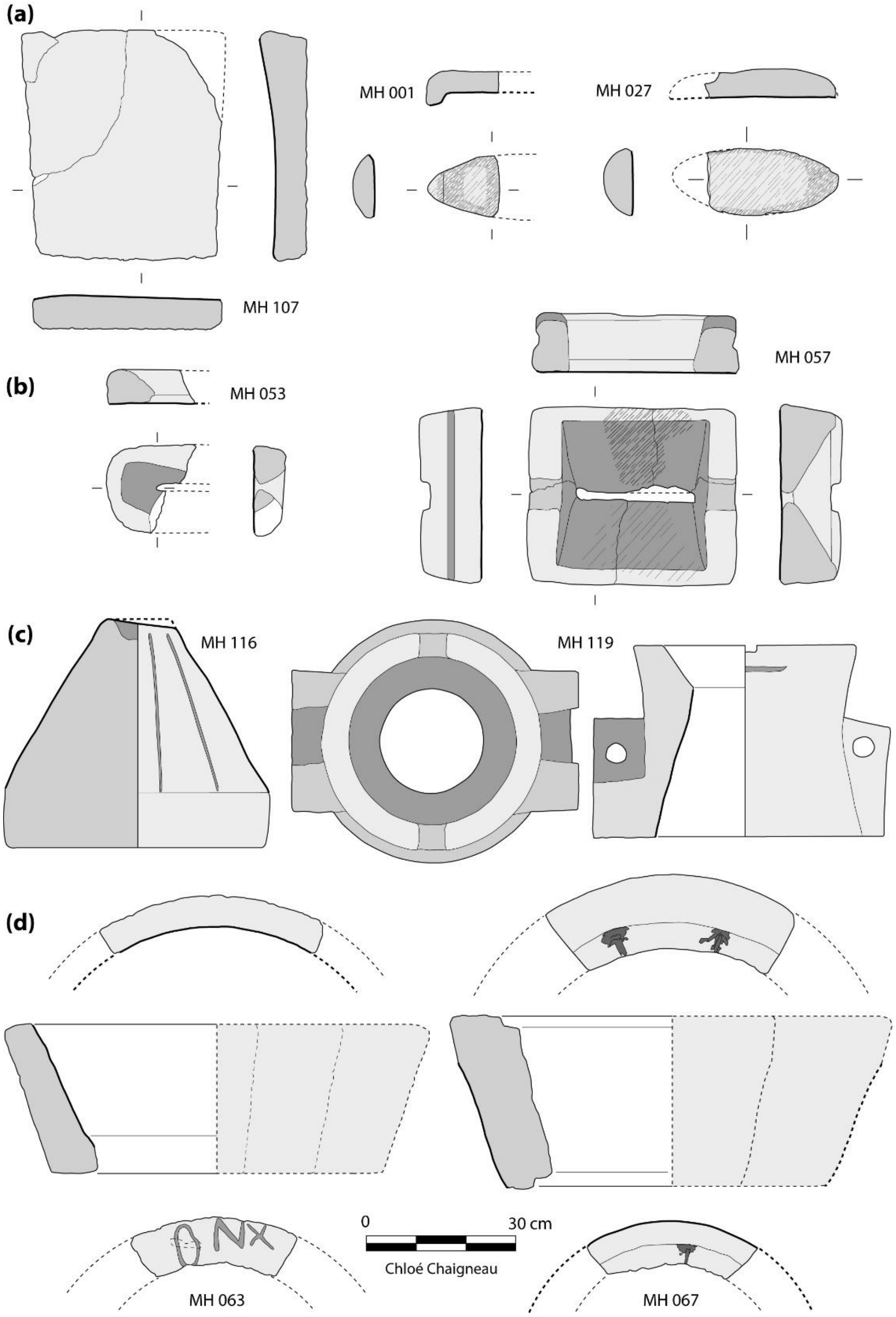

Figure S2 - Thin section petrography of representative volcanic millstone lithotypes by optical polarized microscope at crossed Nicols (a, b, c, e, f) and plane polarized light (d, g, h, i, l). Abbreviations: Gdm: groundmass, Ol: olivine, Pl: plagioclase, Cpx: clinopyroxene, Kfs: K-feldspar, Hbl: horneblende, Lct: leucite, Hyn: haüyne.

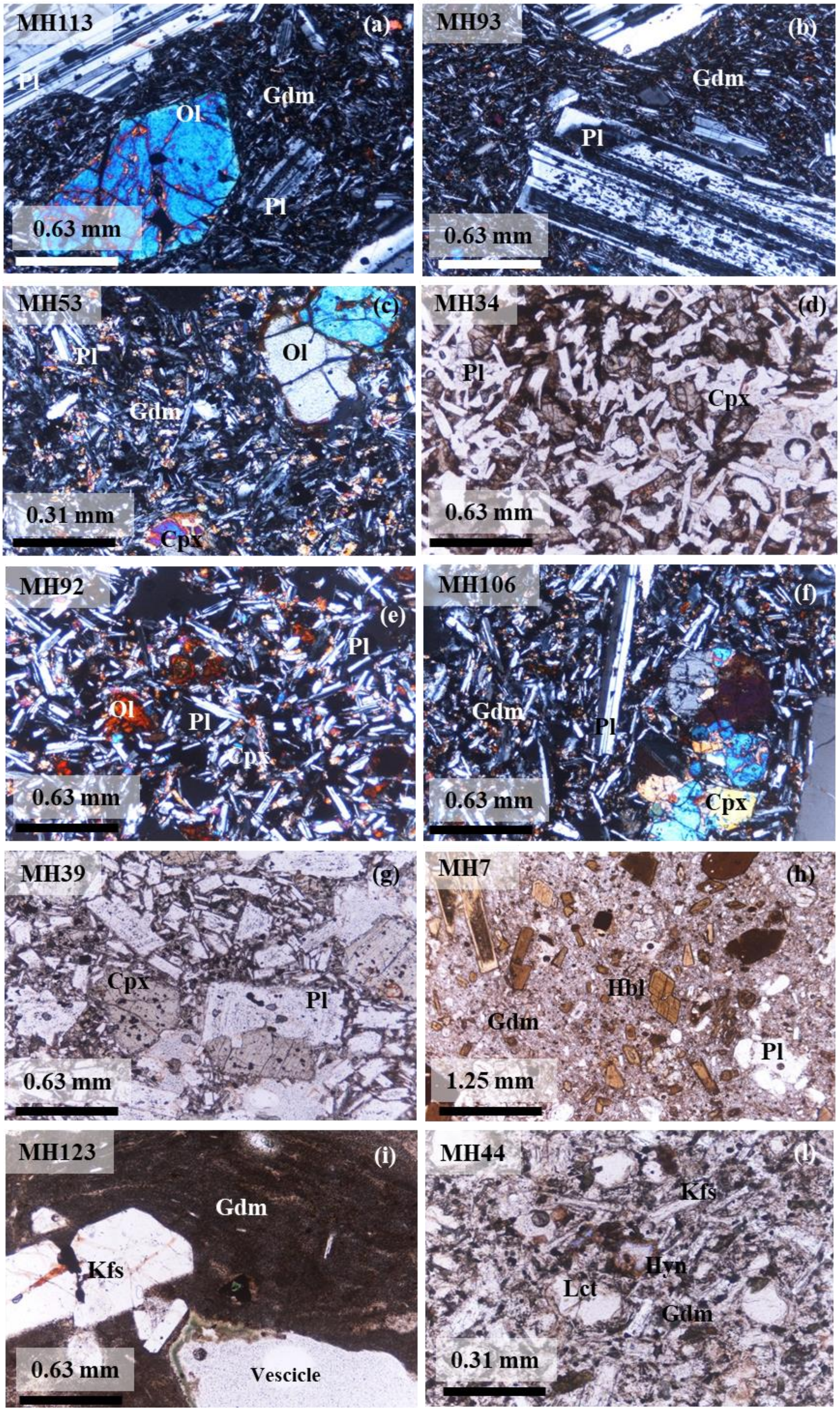

Table S1 - A summary of samples collected in the archaeological site of Megara Hyblaea and their relative composition (classification and magmatic series). Abbreviations: TH = Tholeiitic Series; TH-T = Tholeiitic Transitional Series; Na-ALK = Na-Alkaline Series; CA = Calcalkaline Series.

**Saddle-querns**		**Hopper-rubbers**	
Basal flat portion		MH053	Basalt (TH-T)
MH092	Basaltic andesite (TH)	MH055	Mugearite (Na-ALK)
MH093	Mugearite (Na-ALK)	MH056	Mugearite (Na-ALK)
MH097	Andesite (CA)	MH057	Mugearite (Na-ALK)
MH099	Basaltic andesite (TH)	MH124	Mugearite (Na-ALK)
MH100	Basaltic andesite (TH)	MH125	Mugearite (Na-ALK)
MH104	Basalt (TH-T)		
MH106	Basaltic andesite (TH-T)		
MH107	Basaltic andesite (CA)	**Morgantina-type**	
Upper mobile portion		*Meta*	
MH001	Andesite (CA)	MH113	Mugearite (Na-ALK)
MH004	Basaltic andesite (TH)	MH114	Mugearite (Na-ALK)
MH007	Andesite (CA)	MH115	Basaltic andesite (TH)
MH010	Basaltic andesite (TH)	MH116	Mugearite (Na-ALK)
MH013	Basaltic andesite (TH-T)	MH117	Mugearite Na-ALK)
MH014	Basaltic andesite (TH)	MH118	Mugearite (Na-ALK)
MH024	Basaltic andesite (TH)	*Catillus*	
MH025	Basaltic andesite (CA)	MH074	Mugearite (Na-ALK)
MH026	Basaltic andesite (TH)	MH078	Mugearite (Na-ALK)
MH027	Andesite (CA)	MH119	Basaltic andesite (TH)
MH029	Basaltic andesite (CA)	MH121	Rhyodacite (Ignimbrite; CA)
MH032	Andesite (CA)	MH123	Rhyodacite (Ignimbrite; CA)
MH034	Basaltic andesite (TH)		
MH035	Andesite (CA)	**Delian-type**	
MH037	Basaltic andesite (TH)	MH059	Mugearite (Na-ALK)
MH039	Basaltic andesite (CA)	MH060	Mugearite (Na-ALK)
MH040	Mugearite (Na-ALK)	MH063	Mugearite (Na-ALK)
MH044	Phonolite (HK-ALK)	MH067	Mugearite (Na-ALK)
MH045	Basaltic andesite (TH)	MH068	Mugearite (Na-ALK)
MH052	Andesite (CA)	MH071	Mugearite (Na-ALK)
MH128	Basaltic andesite (CA)	MH072	Mugearite (Na-ALK)
MH129	Basaltic andesite (TH)	MH075	Mugearite (Na-ALK)
MH130	Andesite (CA)	MH120	Mugearite (Na-ALK)
